# Testing the Capability of Embedding-Based Alignments on the GST Superfamily Classification: The Role of Protein Length

**DOI:** 10.3390/molecules29194616

**Published:** 2024-09-29

**Authors:** Gabriele Vazzana, Castrense Savojardo, Pier Luigi Martelli, Rita Casadio

**Affiliations:** Biocomputing Group, Department of Pharmacy and Biotechnology, University of Bologna, 40126 Bologna, Italy; gabriele.vazzana2@unibo.it (G.V.); castrense.savojardo2@unibo.it (C.S.)

**Keywords:** Glutathione S-transferases, protein language models, protein classification, functional annotation, embedding-based alignment

## Abstract

In order to shed light on the usage of protein language model-based alignment procedures, we attempted the classification of Glutathione S-transferases (GST; EC 2.5.1.18) and compared our results with the ARBA/UNI rule-based annotation in UniProt. GST is a protein superfamily involved in cellular detoxification from harmful xenobiotics and endobiotics, widely distributed in prokaryotes and eukaryotes. What is particularly interesting is that the superfamily is characterized by different classes, comprising proteins from different taxa that can act in different cell locations (cytosolic, mitochondrial and microsomal compartments) with different folds and different levels of sequence identity with remote homologs. For this reason, GST functional annotation in a specific class is problematic: unless a structure is released, the protein can be classified only on the basis of sequence similarity, which excludes the annotation of remote homologs. Here, we adopt an embedding-based alignment to classify 15,061 GST proteins automatically annotated by the UniProt-ARBA/UNI rules. Embedding is based on the Meta ESM2-15b protein language. The embedding-based alignment reaches more than a 99% rate of perfect matching with the UniProt automatic procedure. Data analysis indicates that 46% of the UniProt automatically classified proteins do not conserve the typical length of canonical GSTs, whose structure is known. Therefore, 46% of the classified proteins do not conserve the template/s structure required for their family classification. Our approach finds that 41% of 64,207 GST UniProt proteins not yet assigned to any class can be classified consistently with the structural template length.

## 1. Introduction

After the success of Large Language Models (LLMs) for natural language processing tasks, transformer-based deep-learning architectures [1] have taken hold in the field of computational biology, with the consequent emergence of a counterpart adapted to protein sequences, known as protein Language Models (pLMs) [2,3]. Several pLMs have been implemented in the past few years, mainly differing in relation to the number of sequences included in the training set (of particular relevance are the models developed by Rost’s lab [3,4] and the more recent ESM family of models developed by the MetaAI group [5,6]). Recently, pLMs have emerged as a new and powerful mapping procedure which allows the representation of a protein sequence considering the knowledge that the protein can derive from its family and/or superfamily, in the multifaceted protein universe [7]. This procedure, referred to as “embedding”, is “context-aware” [7] and it is often adopted to generate input to train downstream predictive tools with machine and/or deep learning approaches, replacing the classic method based on the time-consuming generation of Multiple Sequence Alignments (MSAs). The embedding procedures have been increasing the performance of relevant predictive tasks, including protein secondary structure [3], protein–protein interaction [8,9] and three-dimensional (3D) protein structure prediction [6]. Different embedding-based methods made it possible to quantify sequence similarity [3,10,11], to cluster proteins into families [12], to generate evolutionary landscapes [13,14] and to search for structure–structure similarities [15,16], just to mention some of the applications.

Summing up, we may conclude that the embedding procedures succeed in carrying along information derived from the protein family/superfamily, including sequence profile and template structure conservation. It is still debatable whether embedding is sufficient to recognise remote homologs and perform functional annotation, as recently discussed [17,18].

Now, in light of these advancements, a key question remains: to which extent can we stress the embedding procedure for sequence alignments? In order to test this, here we tackle the annotation problem of the GST superfamily, with a new method for determining sequence embedding distances, outperforming previous ones in remote homology detection (EBA, embedding-based alignment, [18] and references therein). We choose the Glutathione S-Transferases superfamily (GST, EC 2.5.1.18, [19]) for its functional and structural characteristics. According to the literature, the superfamily includes three major groups, cytosolic, mitochondrial and microsomal, with at least 20 documented families (or classes), active in the different cell compartments. Although these enzymes function in the same cellular compartment, their structure remains conserved despite low sequence identity across classes and different organisms. A total of 75% of the classes share the same functional fold and are active in the cytosol together with the other three structural classes; two other folds are active in mitochondria and in microsomes, respectively [20,21,22,23,24,25,26]. The complex relation between sequence and structure makes the annotation process difficult (see Supplementary S1 [27,28,29,30,31,32,33,34,35,36,37] for an extended description of the classes). 

In the following, we test the capabilities of embedding-based alignment in the task of assigning sequences to the different GST classes as done in UniProt with an automatic procedure defined by the ARBA/Uni rules [38]. After the selection of a reference set, we undertake large-scale testing, adopting the recent MetaAI ESM2-15b pLM and measuring sequence distance with EBA [18]. We find that the procedure is successful in sequence annotation, particularly when the sequence length of the proteins is conserved with respect to those included in the reference set. With this constraint, we classify another 26,180 proteins from 64,207 unclassified GSTs in UniProt, enriching the number of proteins in the different classes, and generating a set of sequences for future experimental investigations.

## 2. Results and Discussion

### 2.1. Fishing for Transfer of Annotation

**Figure 1:**
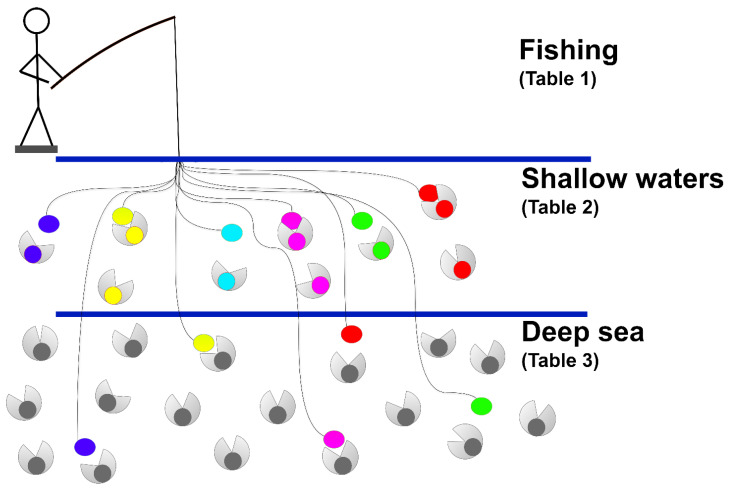
Embedding-based alignment for GST protein classification. A reference protein set of well-curated proteins (described in Table 1) is adopted as baits for “fishing” ARBA GST classified proteins (testing set, in shallow waters, Table 2) and ARBA GST unclassified proteins (in deep sea, Table 3). For details, see text and Section 3. Color matching is indicative of the affinity of baits and preys.

Our procedure is described in Figure 1. Basically, we generate a reference set of the GST protein superfamily which acts as a representative set of the functional and structural properties of the proteins in the superfamily. The set is carefully selected and contains proteins with a reference PDB structure and/or a high-quality AlphaFold2 model, along with an experimental validation of the function. Then, each protein of the reference set is embedded with the selected protein language model and becomes a bait. The encoding procedure allows for carrying information on the structure and on the conserved sequence motifs of the family [2,3,4,5]. The embedded bait is then aligned with the EBA alignment procedure [18], with a testing set from UniProt, filtered with the UniProt/ARBA rules, and annotated in a specific GST class. The different reference classes are color coded in Figure 1, and fishing in shallow waters is successful when a prey with the same color is captured (in this case, the assigned annotation obtained with embedding alignments matches with the one already present in the testing set). Finally, we enter with the procedure into the deep sea, to search for new proteins to add to a specific family. After validation of the procedure in the previous step, we now classify proteins without any verification.

### 2.2. The Reference Set

Our reference set (Ref set) is detailed in Table 1. It contains 284 well-annotated proteins from SwissProt, with documented experimental evidence. When listing per taxon, the presence of specific classes in particular taxon (e.g., Phi, Tau, Lambda only in Viridiplantae, Beta and HSP 26 only in bacteria) is evident. A more detailed description of the GST superfamily is available in Supplementary S1. It appears that, for the time being, four different folds are adopted by GST proteins functionally active in the cytosol; however, the most populated one is conserved for 15 classes, active in the cytosol and collecting proteins that are or are not distantly related homologs (see rightmost columns in Table 1 where the length variability together with the sequence identity range are reported). Considering a 30% sequence identity, the threshold between homologs and distantly related ones, six classes indeed contain distantly related homologs, where the conserved structure in the PDB testifies to the inclusion in the class (family). Interestingly, in Table 1, 245 proteins share the same fold and are distributed in 15 classes. Other folds are present in the cytosol of fungi (omega-like), bacteria (FosA) and mammals (LanC). The length of these proteins is different from the previous ones. Finally, kappa and MAPEG have been reported in mammals and are active in the mitochondria and in the microsomes, respectively. Both classes include remote homologs and folds different from the cytosolic ones. The proteins in the different classes share less than 30% sequence identity (Table S1).

**Table 1:** The reference dataset (REFset) with 284 sequences.

Classes	Bact.	Amoeb.	Fungi	Virid.	Plat.	Nem.	Arth.	Moll.	Actin.	Amph.	Aves	Mamm.	Total Class	Length	Seq. Id. Range (%)	Structure
**Mu**	-	-	-	-	11 (9 *)	-	3 (2 *)	-	-	-	1 (1)	28 (8 *)	43 (20 *)	211–225	22–98	
**Sigma**	-	-	-	-	-	9 (2)	7 (4 *)	1 (1)	-	-	1	3 (2)	21 (9 *)	199–249	25–94	
**Alpha**	-	1	-	-	-	-	-	-	-	-	2 (1)	18 (10 *)	21 (11 *)	222–229	29–96	
**Pi**	-	-	-	-	-	5 (2 *)	-	-	-	2	-	12 (3)	19 (5 *)	207–210	32–99	
**Theta**	-	-	-	-	-	-	-	-	-	-	-	12 (3)	12 (3)	240–244	40–99	
**Delta-Epsilon**	-	-	-	-	-	-	32 (15 *)	-	-	-	-	-	32 (15 *)	208–271	25–99	
**Omega**	-	-	-	-	-	3	2 (2 *)	-	-	-	-	7 (2)	12 (4 *)	240–256	23–93	
**Zeta**	3 (3 *)	-	1 (1)	3 (1)	-	1	-	-	-	-	-	3 (2)	11 (7 *)	212–221	33–95	
**Rho**	-	-	-	-	-	-	-	1 (1 *)	1	-	-	-	2 (1 *)	223–225	41	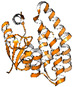
**DHAR**	-	-	-	3 (3)	-	-	-	-	-	-	-	-	3 (3)	213–213	66–76	
**Tau**	-	-	-	34 (5 *)	-	-	-	-	-	-	-	-	34 (5 *)	217–231	30–98	
**Phi**	-	-	-	25 (11 *)	-	-	-	-	-	-	-	-	25 (11 *)	212–221	31–95	(8GSS)
**Lambda**	-	-	-	3	-	-	-	-	-	-	-	-	3	235–237	56–73	
**Beta**	4 (3)	-	-	-	-	-	-	-	-	-	-	-	4 (3)	201–203	36–54	
**HSP26**	3 (3)	-	-	-	-	-	-	-	-	-	-	-	3 (3)	202–212	22–60	
**Omega-like**	-	-	4 (1)	-	-	-	-	-	-	-	-	-	4 (1)	313–370	44–63	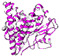 (5LKD)
**FosA**	2 (2)	-	-	-	-	-	-	-	-	-	-	-	2 (2)	135–141	59	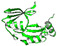 (1NPB)
**LanC**	-	-	-	-	-	-	-	-	1	-	-	4 (1)	5 (1)	399–405	63–96	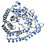 (8D19)
**Kappa**	-	-	-	-	-	2	-	-	-	-	-	3 (2)	5 (2)	225–226	28–86	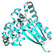 (3RPN)
**MAPEG**	-	1	-	-	-	-	-	-	-	-	-	22 (5)	23 (5)	146–155	12–98	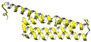 (4AL0)
**Total Taxon**	12 (11 *)	2	5 (2)	68 (20 *)	11 (9 *)	20 (4 *)	44 (23 *)	2 (2 *)	2	2	4 (2)	112 (38 *)	284 (111 *)			

**Legend to** Table 1. The 284 proteins of the reference set are listed according to their classes (rows) and taxonomic groups (columns). The GST superfamily includes four folds in the cytosol and two folds in mitochondria and microsomes, respectively. Cytosolic GSTs comprise 15 classes with the same fold. Three other folds are cytosolic, and two are found in mitochondria (Kappa) and in microsomes (MAPEG), respectively, for a grand total of 20 classes. Taxa are listed, according to the classification adopted in NCBI at phylum (Mollusca, Arthropoda, Nematoda and Platyhelminthes), superclass (Actinopterygii) or class (Mammalia, Aves and Amphibia) for metazoan, at kingdom level for Viridiplantae and Fungi and at superkingdom level for Bacteria. Amoebozoa are also included. The number of proteins with a PDB reference is specified inside round brackets; (*) indicates that at least one entry in the set belongs to TrEMBL. Entries without a PDB reference are endowed with high-quality AlphaFold2 models (see Materials). Dashed horizontal lines discriminate classes in the same sub-cellular location. The “Length” column displays the shortest and the longest protein sequence found in each class. The Seq.Id. (%) column shows the minimum and maximum sequence identity percentage found within each class (for classes with only two representatives, the sequence identity between the two is shown). Abbreviations used: Bact., Bacteria; Am., Amoebozoa; Fu., Fungi; Vir., Viridiplantae; Plat., Platyhelminthes; Nem., Nematoda; Arth., Arthropoda; Moll., Mollusca; Act., Actinopterygii; Amph., Amphibia; Mamm., Mammalia., DHAR, dehydroascorbate reductase; HSP26, Heat Shock protein 26 kDa; FosA, Fosfomycin resistance; and LanC, LanC-like. A close inspection of the literature on the arthropoda proteins indicates that they belong to the delta or epsilon classes, closely related in both sequence and structure [39,40], and we included these proteins in one single delta-epsilon class (as also suggested by the Conserved Domain Database (CDD), https://www.ncbi.nlm.nih.gov/Structure/cdd/cdd.shtml. (accessed on 14 June 2024)). As to the Rho class, we added it after the reclassification of previous theta into rho GST proteins, found in marine organisms [41]. All of the structures shown are from PDB current release (https://www.rcsb.org/ (accessed on 1 January 2024)). From structural alignment we find that the root mean square deviation (RMSD) within class is less than 3 Å.

**Table 2:** Testing the embedding-based alignment (EBA) towards the ARBA GST classification.

	ARBA*	Within Reference Length Range (RLR)*				Below Reference Length Range (< RLR)*					Above Reference Range (>RLR)*				
Class	Total	°Exp	°Pred		°Pred SI*	°Exp	°Pred		°Pred SI*	Errors	°Exp	°Pred		°Pred SI*	Errors
	(#)	(#)	(#)	RLR	(%)	(#)	(#)	<RLR	(%)	(#)	(#)	(#)	>RLR	(%)	(#)
**Mu**	1706	981	979	211–225	10–99	355	349	140–210	8–99	6	370	335	226–475	9–99	35
**Sigma**	694	592	592	199–249	20–99	66	66	109–198	21–99	-	36	36	250–499	3–99	-
**Alpha**	1520	734	734	222–229	17–99	495	471	113–221	13–99	24	291	289	230–487	10–99	2
**Pi**	609	323	323	207–210	24–99	158	158	120–206	21–99	-	128	124	211–488	20–99	4
**Theta**	1428	560	560	240–244	25–99	545	540	104–239	16–99	5	323	323	245–491	1–99	-
**Delta-Epsilon**	822	715	715	208–271	18–99	68	68	102–207	15–99	-	39	39	272–478	10–99	-
**Omega**	1349	556	556	240–256	11–99	617	597	101–239	6–99	20	176	176	257–474	10–99	-
**Zeta**	728	268	268	212–221	26–99	122	122	139–211	22–99	-	338	338	222–433	2–99	-
**DHAR**	10	-	-	213–213	-	7	7	107–212	6–97	-	3	3	214–465	37–99	-
**Tau**	1342	851	851	217–231	23–99	222	219	202–216	6–99	3	269	268	232–449	3–99	1
**Phi**	1711	1066	1066	212–221	25–99	177	177	149–211	20–99	-	468	468	222–491	2–99	-
**HSP26**	433	363	363	202–212	33–99	48	48	196–211	40–99	-	22	22	213–227	40–99	-
**LanC**	450	177	177	399–405	45–99	109	109	126–398	4–99	-	164	164	406–490	32–99	-
**Kappa**	1148	230	230	225–226	17–99	554	554	189–224	12–99	-	364	364	227–257	12–99	-
**MAPEG**	1111	687	687	146–155	7–99	143	143	101–145	3–99	-	281	281	157–363	6–99	-
**Total**	15,061	8103	8101			3686	3628			58	3272	3230			42

**Legend to** Table 2. * The testing set includes 15,061 GST proteins classified by the ARBA rule system [38]. * From Table 1, we derived the reference length range (RLR) of GST proteins with a reference fold per each class present in the set (see Section 3, Table 1). For the sake of fold conservation, we clustered GST proteins as proteins with a length included in the range (RLR), below the range (<RLR) and above range (>RLR). We show also the range of sequence identity per EBA-found GST class (Pred SI). EBA errors are particularly on GST proteins with lower or higher length than those in the range of fold conservation. See text for details and discussion. Only two mu proteins in the reference length range are misclassified by EBA: (UniProt IDs: A0A1I8FWQ8, A0A1I8J3A2) are classified as sigma by the embedding procedure. A sequence comparison of the two proteins with the sigma and mu GST proteins indicates that they share higher sequence identity with the sigma than with mu GST reference proteins (33% and 28% sequence identity, respectively). Moreover, the sigma classification is supported by the presence of CDD sigma domains in the annotation. °Exp = ARBA expected; Pred = EBA classification. Pred SI = Sequence identity among predicted and reference class (Table 1); # = Number of. In the testing dataset, among the mu-class GSTs, a set of 29 similar sequences (sequence identity >40%) above the reference length range are misclassified. InterPro annotations for this group of proteins reveal the presence of canonical GST domains together with an extra Elongation Factor 1B domain, suggesting that the canonical fold is not conserved. Most of the remaining errors are found in the “below reference length range” region of the alpha and omega classes. Among these, 30 are due to the normalization procedure of the method, as the similarity alignment score (s_align) is higher for the correct class. Indeed, when a test protein is shorter than the representative entries of the class in the reference dataset, the ${EBA}_{min}$ score for the correct classification is penalized with respect to shorter sequences of a different class, possibly resulting in misclassifications. In the case of omega errors they are always classified as tau, with the former class showing longer sequences in the reference dataset with respect to the latter. Interestingly, these two classes show deep structural similarities, with the tau class lacking an N-terminal extension typical of omega cGSTs [42]. The remaining misclassifications are either normalization-derived or driven by sequence similarity with the bait proteins.

**Table 3:** Classifying GST proteins in the “deep sea” with the embedding-based alignment method.

Class	Bacteria	Amoeb.	Fungi	Virid.	Plat.	Nematoda	Arth.	Moll.	Actin.	Amph.	Aves	Mamm.	Others	Total Class
**Mu**	5	-	33	4	6	12	74	7	5	-	-	7	96	249
**Sigma**	30	-	87	14	-	480	133	82	11	3	73	130	376	1419
**Alpha**	21	-	13	7	-	11	1	1	2	-	1	6	49	112
**Pi**	-	-	37	2	-	6	4	-	-	1	-	4	33	87
**Theta**	13	-	-	10	-	-	1	3	2	-	-	30	5	64
**Delta-Epsilon**	1949	9	498	8	-	-	1642	1	5	-	-	4	74	4190
**Omega**	87	-	112	5	1	-	1	-	-	-	-	-	10	216
**Zeta**	1397	-	22	7	-	-	-	-	-	-	1	-	21	1448
**Rho**	524	-	22	-	-	-	-	5	197	-	-	-	9	757
**DHAR**	1	-	-	-	-	-	-	-	-	-	-	-	-	1
**Tau**	1555	-	30	2401	-	1	-	-	1	-	-	-	41	4029
**Phi**	3694	5	772	60	-	-	-	1	1	-	-	1	57	4591
**Lambda**	-	-	3	63	-	-	-	-	-	-	-	-	-	66
**Beta**	1569	-	4	-	-	-	1	-	-	-	-	-	15	1589
**HSP26**	2539	-	9	1	-	-	-	-	-	-	-	-	27	2576
**Omega-like**	2746	1	298	39	-	-	2	-	4	-	2	21	200	3313
**FosA**	306	-	-	-	-	-	-	-	-	-	-	-	2	308
**LanC**	-	-	-	-	-	-	-	-	-	-	-	-	1	1
**Kappa**	-	-	8	-	-	-	-	-	1	-	-	-	-	9
**MAPEG**	39	1	230	108	3	-	347	24	141	11	48	44	159	1155
**Within RLR**	16,475	16	2178	2729	10	510	2206	124	370	15	125	247	1175	26,180
**Below RLR**	11,288	6	626	1493	66	237	443	52	426	39	159	509	677	16,021
**Above RLR**	12,917	22	3743	2260	18	119	388	45	342	15	56	148	1007	21,080
**Total per Taxon**	41,075	44	6582	6851	99	883	3063	222	1157	69	345	928	2889	64,207

**Legend to** Table 3. The trial set contains 64,207 GST ARBA unclassified proteins. The EBA method classifies 26,180 proteins whose range of lengths (within reference length range (RLR)) ensures structure conservation with respect to baits, about 41% of the total. A total of 58% is classified below and above the range (below and above RLR), and another 1% is not classified. The spreading of the classes in different taxa from those in Table 1 is discussed in the text. The trial dataset contains more bacteria genera (700) than the reference and testing dataset (200). Proteins classified in the reference length region belong to plant symbionts (Rhizobium, Sinorhizobium and Rhizobiales), plant pathogens (Acidovorax), photosynthetic bacteria (Synechoccus and Nostoc) and soil bacteria (Acinetobacter, Azospirillum, Myxococcus, Streptomyces, Sphingomonas and Variovorax). The classification led to the enrichment of new GST classes for bacteria not found in the reference and testing datasets. As an example, a couple of “new” alpha-class bacterial Myxococcus proteins (UniProt IDs: F8CAS1, Q1D6B3) share 34% sequence identity with alpha-class proteins in the reference dataset. More functional and structural studies are necessary for data validation.

### 2.3. Testing the Embedding Alignment Method

After embedding the reference proteins, we tested the EBA procedure (see Section 3) to classify the protein of the testing set, already classified by the UniRule/ARBA automatic annotation system of UniProt. Results are shown in Table 2. The main difference between ARBA and EBA is that ARBA classifies after finding conserved domains and/or motifs that are typical of the GST superfamily, without any constraint on the sequence length of the protein, while EBA considers the pairwise global alignment of any two protein embedded sequences. One should also consider that 15 different classes of the canonical cytosolic GSTs share the same fold, and therefore the same InterPro domains. This makes their classification difficult, considering that within classes remote homologs are also present. In this respect, Table 1 lists our baits along with the length range associated with the different folds, and their range of sequence identity. Since all of the baits are complete proteins, we follow the knowledge that a transfer of classification is reliable when the protein fold is conserved [43], and this implies that the protein length is conserved. Accordingly, in Table 2, we divide EBA-classified GST proteins into three groups: within, below and above the length range of fold conservation. This division identifies the fraction of the GST proteins (within the range of length of the of the baits) which conserve the structure. In this subset, the number of remote homologs with respect to the reference set is 76 (five in mu, three in sigma, thirteen in omega and fifty-five in kappa, see https://bar.biocomp.unibo.it/GST_Datasets/index.htm (accessed on 19 September 2024). Out of the 3D conservation range, the method is any way successful (Table 2). Overall, the prediction accuracy of our method with respect to ARBA in classifying proteins is very high (99.3%). Interestingly enough, it appears that the differences in classification between our method and ARBA rules in Table 2 are mainly confined in regions below and above the range of fold conservation (Table 2), when the protein length is lower or higher than those of the reference set. A closer inspection indicates that in these regions, the predicted protein can be included into the prey (lower length) or can include it (higher length). In other words, in these regions, the embedding-based alignment captures domains and motifs like the ARBA rules. Proteins in the below regions contain fewer motifs/domains than the prey while in the above regions they include extra motifs/domains. This is consistent with the notion that structure is not conserved; therefore, the definition of remote homologs fails. In these regions, errors are mainly due to the fact that baits from other classes share a higher identity with the GST protein at hand. Results are also detailed by taxon (Table S2). 

### 2.4. Fishing in the Deep Sea

We tried EBA to classify 64,207 ARBA-unclassified GST proteins (Table 3). We show only results obtained on GST proteins whose length is in the length range of fold conservation and classify 41% of the total. Out of the safety range we can transfer class to another 58% GST proteins. The range of classes seems to increase, particularly in GST proteins from bacteria, and this can be explained by considering the new bacterial genomes recently included in TrEMBL. However, more functional and structural studies are necessary for data validation. Setting a length interval for structure conservation highlights more reliable predictions.

## 3. Materials and Methods

### 3.1. Dataset Generation

In order to address our task, we downloaded three different datasets from UniProt (release 2024_01, https://www.uniprot.org/ (accessed on 24 January 2024)).

#### 3.1.1. The Reference Dataset

We collected all of the GST proteins including “Glutathione S-transferase” and/or “Glutathione transferase” in the protein name, endowed with a PDB structure or a high-quality AlphaFold2 model [44], and excluding fragments. For each of the proteins linked to a PDB structure, we selected a representative based on resolution, sequence coverage (higher than 70%) and, when possible, in complex with the glutathione substrate. We checked that proteins lacking PDB structures are endowed with high-quality AlphaFold2 models (with a per protein average pLDDT (predicted Local Distance Difference Test) value ≥ 70 [44]), whose root mean square value (RMSD) to the backbone of the 3D representatives of the class is ≤1.5 Å. We retained 284 proteins (Table 1), characterized by six structural types, and grouped into 20 GST classes. We also grouped GST reference proteins in relation to their taxa (https://www.ncbi.nlm.nih.gov/taxonomy (accessed on 15 June 2024), [45]). The reference dataset is available at https://bar.biocomp.unibo.it/GST_Datasets/index.htm (accessed on 19 September 2024).

#### 3.1.2. The Testing and Trial Datasets

With a similar search we collected all of the TrEMBL (https://www.uniprot.org/ (accessed on 24 January 2024) sequences named “Glutathione S-transferase” and/or “Glutathione Transferase”. Routinely, the protein name is automatically assigned, together with the Enzyme Commission (EC) number, when the sequence entry annotation satisfies either UniRule ID UR000000494 or ARBA ID ARBA00012452, respectively (https://www.uniprot.org/help/arba (accessed on 24 January 2024), [38,46]). The rules are routinely based on the automatic recognition of GST-specific motifs and/or domains in the sequence. In some cases, ARBA rules, satisfying class-specific InterPro [47] signatures, assign a specific class to the protein (ARBA rules are present for 14 of the 20 classes, https://www.uniprot.org/arba (accessed on 19 September 2024) (see above)). After filtering out all of the entries with a “Caution” statement in the “Function” field of the protein file and proteins with sequence identity values higher than 95% to the reference set, we retained a testing set with 15,061 GST proteins annotated with an assigned class and a trial set with 64,207 GST proteins without classification.

### 3.2. Embedding Procedure

#### 3.2.1. Embedding Generation

Among the pLMs currently available, the MetaAI ESM2 encoding set has been used to train ESMFold [6], an advanced protein tertiary structure prediction method ([48], and references therein). We adopt the most recent ESM2 pLM: ESM2-15b trained on 65 million proteins [6]. For each protein in the three datasets, we extracted the ESM2-15b representations following the instructions and scripts available at https://github.com/facebookresearch/esm (accessed on 1 November 2022). Given an input protein sequence of length l, the pLM outputs meaningful distributed vector representations for each amino acid residue of the protein at hand. The size D of the vectors depends on the number of hidden states of the transformer layers from which the representations are extracted (routinely the last one). ESM2-15b outputs vectors with D=5120. The final encoding of the protein is therefore a matrix $e\;\in R^{l\times D}$, (with *l* as the protein length), routinely referred to as per-residue protein embedding. 

#### 3.2.2. Embedding-Based Alignment

We compared per-residue GST protein embeddings exploiting the embedding-based alignment (EBA) method [18]. The algorithm (available at https://git.scicore.unibas.ch/schwede/EBA (accessed on 1 January 2023)) computes a pairwise distance matrix of per-residue embeddings, evaluating the Euclidean distance of all embedded residues. These values fill a matrix of dimension *l*_1_ × *l*_2_ (where *l*_1_ and *l*_2_ are the lengths of the two proteins, respectively), which provides the substitution scores for the pairwise alignment based on a classic dynamic programming approach. The tool includes also an optimizing intermediate step, called “signal enhancement” [18], where each score of a residue pair is normalized to the scores of all residue pairs of the two aligned proteins. We adopt this enhanced similarity matrix to score the pairwise global alignment obtained with the Needleman–Wunsch (NW) method [18]. Following the procedure, we normalize the alignment similarity score $s_{align}$ by the length l of the longer sequence in the pair ($l_{max}$), according to the following:


$${EBA}_{min}=\frac{s_{align}}{l_{max}}$$

Following [18], the length normalization is an important factor on the final score when the proteins being compared are very different in length. Whenever the difference is large, a high ${EBA}_{max}$ score, obtained by normalizing by the length of the shortest protein, reflects the fact that the shorter sequence is entirely contained in the longest [18]. In this case, ${EBA}_{min}$ is much lower since the longer sequence is only partially aligned. Following the author’s suggestion and considering template structure conservation as an essential element of knowledge transfer [43], we adopted the ${EBA}_{min}$ to score any two protein sequences during our procedure. In any case, each protein after embedding is aligned with all of the other ones. For a given query protein, we compute ${EBA}_{min}$ by aligning to all of the proteins in the reference set. The query protein assumes the class annotation of the best scoring protein among the references and then classification (annotation) is transferred. By this a bait can “fish” a prey (Figure 1). 

The main difference of our method, as compared to [18], is the adoption of ESM2-15b with an embedding vector dimension of 5120 and a different procedure for the output selection (ProstT5 [4] with a vector dimension of 1024 was adopted in the original implementation [18]). We analyze results considering that the transfer of knowledge requires structure template conservation [43] and for this reason, present the results as a function of the protein length. 

### 3.3. Computational Time

After downloading EBA in house, the time required to align 100,000 proteins with our reference set (284) was one week with a machine endowed with 80 CPUs and a 754 gigabyte RAM. 

## 4. Conclusions and Perspectives

In this paper, we exploit the capabilities of an embedding-based alignment method [EBA, 18] to annotate proteins like the UniProt ARBA system of automatic annotation. For this, we focused on the GST protein superfamily, given the complex relationship among sequence and structure in the different protein classes which in different taxa characterize the group. GST main characteristics include sequences of different lengths, sharing the same folding when active in the same cellular compartments (Table 1). This blurs the classification of GST proteins in newly sequenced proteomes. The UniProt ARBA automatic annotation system annotates into GST classes proteins of any length, provided that InterPro motifs and/or domains are conserved, without taking into consideration the fold conservation, which obviously sets a limit for the protein length. We find that EBA performance compares well with the ARBA rule annotation system (over 99% of accuracy), and from error analysis (Table 2), we derive as a rule of thumb that classification is optimal when fold is also conserved. We find that at least 46% of GSTs of a selected subset of classified TrEMBL GST proteins do not conserve the length of the reference protein folding typical of the class. These proteins, beyond the conservation of the typical GST domains, are often endowed with other domains, possibly suggesting new folds, not yet experimentally available. EBA routinely does not misclassify protein fold, and as to misclassification within a class, it may happen that when the bait is contained or contains the prey, matrix alignment is not sufficient to recognize the subdomain. In this case, sequence alignment to another close class can prevail over the ARBA annotation. The EBA assignment in the fold conservation region is able to recover remote homologs in four classes (mu, sigma, omega and kappa), confirming the capability of the system to also assign to a class/family proteins sharing low sequence identity [18]. We also classify proteins not yet classified in UniProt, releasing a list of proteins for experimental validation. This can encourage experiments to further cluster GST proteins in more functional classes, for a better definition of their role in cell complexity. We propose the EBA classification procedure as a valid complement to the ARBA rule classification system for the GST superfamily, considering that sequence embeddings carry along information on structural templates, motifs and domains of the family.

## Data Availability

The data underlying this article are available in the article, in its online Supplementary Material, and at https://bar.biocomp.unibo.it/GST_Datasets/index.htm (accessed on 19 September 2024).

## Supplementary Materials

The following supporting information can be downloaded at: https://www.mdpi.com/article/10.3390/molecules29194616/s1, Supplementary S1: The GST superfamily; Table S1: Interclass sequence identity between proteins in the reference set; Table S2: Embedding based GST classification on the ARBA Test set.
