# Supplementary material for: Testing the Capability of Embedding-Based Alignments on the GST Superfamily Classification: The Role of Protein Length
Source: Molecules. 2024 Sep 29;29(19):4616. doi: 10.3390/molecules29194616 (PMC11478096; doi:10.3390/molecules29194616)
Supplement: Supplementary file 1 [file molecules-29-04616-s001.zip › molecules-3221979-supplementary.pdf]

Supplementary S1

The GST superfamily

Cytosolic GSTs. The GST superfamily includes different classes (families). "Canonical" GSTs (cGSTs) are cytosolic proteins sharing the same fold (within a maximum Root Mean Square deviation (RMSD) of 3.5 Å) and are routinely included into several families (named also classes) introduced on the basis of sequence similarity, structural properties and chromosomal organization in different organisms [19]. The names of the families are indicated with greek letters. In humans a total of seven families are present: mu, sigma, alpha, pi, theta, omega, zeta. Canonical GSTs are small proteins (roughly 200-250 residues) with a recognizable fold; a N-terminal thioredoxin-like (βαβαββα) and an all-alpha C-terminal domain connected by a short linker [27]. The N-terminal domain is the most conserved among cGSTs and contains the glutathione activating cysteine, serine or tyrosine residues. The functional enzymes exist either as homo or heterodimeric protein, and only subunits within the same class can form heterodimers.

Among the various cGST classes, some are widespread in nature (zeta, theta) while others are often considered taxon specific. Delta and epsilon classes are insect specific [28]; these have high implications on DDT resistance and recent findings suggest the presence of the delta class in other arthropods [39]. The rho class, firstly described in [29], seems to be a unique cGST specific of marine organisms. A set of cGSTs are considered plant-specific (DHAR, tau, phi, lambda [23]), although recent findings suggest the presence of the phi class in fungi and bacteria [30]. Finally, the beta class, firstly found in *Proteus mirabilis*, is bacteria specific [31].

In [32], a novel cytosolic GST protein in Saccharomyces cerevisiae was assigned to the omega class given its ability to use some substrates of the omega class GST. Evidence carried out in [21, 33] suggests that the protein should be considered a new GST structural class. Indeed, it shows very low sequence identity with others omega GSTs and is enriched with additional structural features: a long N-terminal coil (77 residues), a loop between the strand beta2 and alpha2 (30 residues) and a final 20 residues C-terminal coil. Altogether, the protein is much longer than a classical cGST, including more than 300 residues. In [21], this new class is named Xi-class, but this notation is not adopted by UniProt, where the proteins are called "omega-like".

In bacterial cytosol, fosfomycin-resistance GSTs (short proteins of 130-140 residues active as homodimers) have a different fold from cGSTs [31].

Additionally, enzymatic assays on mammalian LanC-like protein 1 (LANCL1) demonstrated that this divergent superfamily, mainly recognized as peptide-modifying enzymes, can catalyse the conjugation of GSH to synthetic substrates, similarly to GSTs [22]. These are cytosolic proteins in physiological conditions [34] and are longer than cGSTs, with 400 or more residues.

Mitochondrial GST

Mitochondrial GSTs (the kappa class) are mostly found in the mitochondrial matrix and peroxisomes [24]. Similarly to cGSTs, kappa GSTs are soluble proteins including ~200 residues. The subunits are organized into homodimers and contain a thioredoxin-like and all-alpha domains. However, the two domains are rearranged with respect to the cGST fold: the all-alpha domain is placed between the beta-alpha-beta and the beta-beta-alpha motifs of the thioredoxin-like domain [25]. As a result the overall structure of the two GST families is quite different and, given the domain sharing, a parallel evolution hypothesis has been proposed [35].

Microsomal GSTs

Microsomal GSTs are members of the MAPEG protein group (Membrane-Associated Proteins in Eicosanoid and Glutathione metabolism) [36]. As membrane proteins they assume a different fold and are shorter than cGST (~150 residues), with subunits organized in trimers [26].

Overall, GSTs are characterized by the presence of remote homologous among structural classes, in particular canonical and MAPEG GSTs. As stated previously, cGSTs are split into at least 15 classes that share the same fold but display very diverse sequences. Indeed, cGST belonging to different classes share less than 30% sequence identity, falling in the so-called twilight region [37]. MAPEG GSTs represent an extreme case, as the sequence identity between two microsomal proteins can drop below 20% sequence identity.

For references see main text references.

Table S1
Interclass sequence identity between proteins of the reference set.

	Mu	Sigma	Alpha	Pi	Theta	Delta-Epsilon	Omega	Zeta	Rho	DHAR	Tau	Phi	Lambda	Beta	HSP26	Omega-like	FosA	LanC	Kappa	MAPEG
**Mu**		17-28	14-27	22-28	11-23	10-22	9-19	10-23	12-20	14-22	7-21	10-26	9-18	11-21	9-20	8-18	6-16	5-14	4-14	1-15
**Sigma**	17-28		16-28	20-28	9-20	11-25	12-23	11-24	11-18	12-20	9-24	11-25	11-20	11-20	12-18	11-21	4-16	5-16	7-17	2-19
**Alpha**	14-27	16-28		19-28	11-19	12-25	8-20	11-28	10-17	11-19	9-20	15-26	10-18	13-22	12-20	11-17	5-15	8-15	6-15	4-19
**Pi**	22-28	20-28	19-28		13-22	11-25	12-22	12-25	15-21	15-23	10-20	11-27	11-17	10-21	9-20	12-19	7-16	6-15	5-16	1-21
**Theta**	11-23	9-20	11-19	13-22		15-26	10-19	17-27	17-21	11-18	13-23	18-26	14-23	15-27	15-18	13-20	6-11	10-14	10-18	6-21
**Delta-Epsilon**	10-22	11-25	12-25	11-25	15-26		13-23	17-27	19-28	14-22	13-27	15-28	13-23	15-23	13-24	13-21	8-21	7-17	10-21	5-25
**Omega**	9-19	12-23	8-20	12-22	10-19	13-23		14-25	14-21	14-25	17-27	11-23	19-25	12-20	12-21	11-20	4-11	8-15	5-19	4-23
**Zeta**	10-23	11-24	11-28	12-25	17-27	17-27	14-25		18-28	16-26	16-26	15-28	16-24	18-26	19-26	15-22	10-19	7-14	9-18	2-19
**Rho**	12-20	11-18	10-17	15-21	17-21	19-28	14-21	18-28		19-24	14-23	18-28	13-18	17-19	18-21	14-15	6-9	7-13	10-15	2-16
**DHAR**	14-22	12-20	11-19	15-23	11-18	14-22	14-25	16-26	19-24		15-24	17-25	19-23	15-19	20-23	13-20	4-16	5-14	10-16	4-20
**Tau**	7-21	9-24	9-20	10-20	13-23	13-27	17-27	16-26	14-23	15-24		12-26	16-28	11-23	18-28	13-21	8-20	6-17	5-15	3-21
**Phi**	10-26	11-25	15-26	11-27	18-26	15-28	11-23	15-28	18-28	17-25	12-26		14-26	14-27	14-25	14-22	6-16	6-17	7-18	4-23
**Lambda**	9-18	11-20	10-18	11-17	14-23	13-23	19-25	16-24	13-18	19-23	16-28	14-26		15-19	15-20	20-25	7-9	11-13	9-14	2-18
**Beta**	11-21	11-20	13-22	10-21	15-27	15-23	12-20	18-26	17-19	15-19	11-23	14-27	15-19		14-24	14-19	9-13	2-8	9-17	3-14
**HSP26**	9-20	12-18	12-20	9-20	15-18	13-24	12-21	19-26	18-21	20-23	18-28	14-25	15-20	14-24		11-16	11-18	8-13	6-16	3-16
**Omega-like**	8-18	11-21	11-17	12-19	13-20	13-21	11-20	15-22	14-15	13-20	13-21	14-22	20-25	14-19	11-16		4-9	10-15	11-17	6-18
**FosA**	6-16	4-16	5-15	7-16	6-11	8-21	4-11	10-19	6-9	4-16	8-20	6-16	7-9	9-13	11-18	4-9		5-9	6-11	3-26
**LanC**	5-14	5-16	8-15	6-15	10-14	7-17	8-15	7-14	7-13	5-14	6-17	6-17	11-13	2-8	8-13	10-15	5-9		6-14	8-19
**Kappa**	4-14	7-17	6-15	5-16	10-18	10-21	5-19	9-18	10-15	10-16	5-15	7-18	9-14	9-17	6-16	11-17	6-11	6-14		7-23
**MAPEG**	1-15	2-19	4-19	1-21	6-21	5-25	4-23	2-19	2-16	4-20	3-21	4-23	2-18	3-14	3-16	6-18	3-26	8-19	7-23	

The matrix shows the minimum and maximum sequence identity between GST classes of the reference set.

Table S2

Embedding based GST classification of the ARBA Test set

	Bact.		Amoeb.		Fungi		Virid.		Plat.		Nematoda		Arth.		Moll.		Actin.		Amph.		Aves		Mamm		Others		Total Class	
**Classes**	**Exp**	**Pred**	**Exp**	**Pred**	**Exp**	**Pred**	**Exp**	**Pred**	**Exp**	**Pred**	**Exp**	**Pred**	**Exp**	**Pred**	**Exp**	**Pred**	**Exp**	**Pred**	**Exp**	**Pred**	**Exp**	**Pred**	**Exp**	**Pred**	**Exp**	**Pred**	**Exp**	**Pred**
**Mu**	-	-	1	1	-	-	1	1	178	137	-	-	161	161	20	20	250	250	20	20	77	77	831	829	167	167	1706	1663
**Sigma**	-	-	-	-	-	-	1	1	-	-	-	-	693	693	-	-	-	-	-	-	-	-	-	-	-	-	694	694
**Alpha**	-	-	36	26	-	-	-	-	-	-	6	2	9	3	7	5	207	206	23	23	351	351	682	679	199	199	1520	1494
**Pi**	-	-	-	-	-	-	1	1	-	-	53	50	4	4	24	24	131	131	30	30	-	-	308	308	58	57	609	605
**Theta**	-	-	-	-	-	-	170	168	1	1	-	-	253	250	9	9	357	357	16	16	104	104	395	395	123	123	1428	1423
**Delta-Epsilon**	-	-	-	-	-	-	-	-	-	-	-	-	822	822	-	-	-	-	-	-	-	-	-	-	-	-	822	822
**Omega**	-	-	-	-	-	-	-	-	21	21	103	93	278	275	72	70	271	268	32	32	55	55	384	384	133	131	1349	1329
**Zeta**	157	157	-	-	25	25	517	517	-	-	-	-	-	-	-	-	-	-	-	-	-	-	-	-	29	29	728	728
**DHAR**	-	-	-	-	-	-	10	10	-	-	-	-	-	-	-	-	-	-	-	-	-	-	-	-	-	-	10	10
**Tau**	-	-	-	-	-	-	1342	1338	-	-	-	-	-	-	-	-	-	-	-	-	-	-	-	-	-	-	1342	1338
**Phi**	-	-	-	-	63	63	1638	1638	-	-	-	-	-	-	-	-	-	-	-	-	-	-	-	-	10	10	1711	1711
**HSP26**	433	433	-	-	-	-	-	-	-	-	-	-	-	-	-	-	-	-	-	-	-	-	-	-	-	-	433	433
**LanC**	-	-	-	-	-	-	-	-	-	-	-	-	8	8	1	1	160	160	12	12	58	58	161	161	50	50	450	450
**Kappa**	-	-	-	-	445	445	3	3	1	1	63	63	38	38	16	16	216	216	16	16	50	50	232	232	68	68	1148	1148
**MAPEG**	-	-	-	-	-	-	-	-	-	-	-	-	-	-	-	-	347	347	26	26	109	109	531	531	98	98	1111	1111
**Total taxon**	590	590	37	27	533	533	3683	3677	201	160	225	208	2266	2254	149	145	1939	1935	175	175	804	804	3524	3519	935	932	15061	14959

The embedding based alignment method was adopted to classify the testing set including 15,061 GST proteins classified by the ARBA rule system (https://www.uniprot.org/arba?query=*). Results are detailed as a function of the different taxa. Accuracy of EBA towards ARBA classification is 99.3%. See text for discussion. Exp=ARBA expected; Pred=EBA classification.
